# ISEED: an always-on cognitive architecture for experience-driven interaction-oriented humanoid robots

**DOI:** 10.3389/fnbot.2026.1920808

**Published:** 2026-09-17

**Authors:** Dario Pasquali, Francesco Rea, Pietro Morasso, Giulio Sandini, Alessandra Sciutti

**Affiliations:** COgNiTive Architectures for Collaborative Technologies (CONTACT) research unit, Italian Institute of Technology (IIT), Genova, Italy

**Keywords:** always-on cognitive architecture, artificial cognition, embodiment, experience-driven learning, human-robot interaction

## Abstract

Current cognitive architectures for social robots, even when conceived as autonomous and adaptable, remain largely grounded in fully digital, von Neumann–style paradigms, where acquired knowledge is detached from individual experience. This contrasts with biological cognition, in which learning emerges from continuous, embodied interaction which leads to personal, non-copyable competencies. In this perspective, we argue that achieving truly adaptive and socially situated robotic agents requires a paradigm shift toward hybrid, bio-inspired, always-on architectures, where dynamic, and digitally implementable components coexist. Such architecture supports continuous activity, structural plasticity, and experience-dependent learning, enabling knowledge to emerge and strengthen from distributed interactions rather than from copiable parameters. We outline a roadmap in which embodiment, dynamical systems, endogenous values, and socially grounded learning mechanisms are central design principles of such cognitive architecture, and we discuss how these ideas can scale beyond sensorimotor skills toward what we believe being important for social robots. These principles are currently being explored in a bioinspired initiative (iCog) aimed at developing the ISEED (Interaction-oriented Situated Embodied Experience-Driven) cognitive architecture, a framework for humanoid social robots, meant to be a testbed for implementing and validating a hybrid, experience-driven approach for real-world cooperative robot partners.

## 1 Introduction

Autonomous robots deployed in real-world, dynamic, social environments must continuously learn from and adapt to changing surroundings, the people within them, and evolving social dynamics. Recently, we witnessed an accelerating pursuit of Artificial General Intelligence for robotics, with foundation models leading to substantial advances in perception, reasoning and embodied interaction. Physical AI increasingly emphasized closed-loop coupling among computation, embodiment, and the environment (Ray, 2025), while modern Vision-Language-Action models integrate multimodal perception, language, and action within generalist policies that can be adapted across tasks and robotic platforms (Ma et al., 2026). Memory-augmented agents can retrieve prior knowledge and reorganize stored facts (Wu and Shu, n.d.; Xu et al., 2025), continual- and lifelong- learning methods allow robots to preserve and recombine capabilities across successive tasks (Meng et al., 2025), and world models support online learning and planning through internally simulated action outcomes (Ding et al., 2025; Wu et al., 2023). Agentic (Bandi et al., 2025) and active-inference architectures (Szadkowski and Faigl, 2025) further combine memory, planning, feedback, prediction, and adaptive control, significantly narrowing the gap between conventional AI and cognitive robotics.

Nevertheless, most of these approaches remain built upon foundation models, with the assumption that increasingly large datasets and models will enable open-ended adaptation (Mon-Williams et al., 2025; NVIDIA, 2025; Firoozi et al., 2025), with little guarantee that such knowledge will generalize to the specific nuances of real-world situations. Also, scaling data collection and model size is already raising environmental (Liu and Yin, 2024; Simon, 2026), economic (Kharpal, n.d.), and societal (X, Email, and LinkedIn, n.d.; Agarwal, 2026) concerns. Such systems are also predominantly designed and evaluated around externally specified tasks, rather than being driven by the agent continuous adaptation to its own environment and needs. Finally, despite progress in explainability and online adaptation, their internal mechanisms remain less transparent and intelligible than those of biological cognitive agents: generated explanations do not necessarily reveal the processes that produced a decision, while local adaptation does not necessarily reorganize the system as a whole. As a result, LLM-based robotic systems remain profoundly different from biological cognitive agents (Sandini et al., 2024; Quattrociocchi et al., 2025; Kadambi et al., 2026). These “epistemological fault lines” (Quattrociocchi et al., 2025) hamper mutual understanding and limit the possibility for a non-expert user to predict the robot’s behavior, to generate fact-supported motivations for the adaptation, and to teach the robot new competences in context-sensitive ways. We posit that these limitations are determined by the lack of the continuous, adaptive and developmentally grounded nature of cognitive agents.

We argue that successful adaptation to dynamic social environments depends on the emergence of personal, embodied, situated knowledge grounded in first-hand experience. In relation to Artificial Intelligence, we refer to this process as Artificial Cognition (ACo): a self-developed form of knowledge that supports the agents’ ongoing, survival-oriented adaptation (Sandini et al., 2024; Sandini et al., 2025). From this perspective, autonomous robots should not rely only on static encyclopedic knowledge, but also on a cognitive architecture that enables the gradual development of ontogenetic capabilities (Sandini et al., 2024; Vernon, 2014). A wide range of cognitive architectures has been proposed to provide such developmental scaffolding (Kotseruba and Tsotsos, 2020), including symbolic architectures like *SOAR* (Laird et al., 1987) and *ACT-R* (Anderson et al., 1997), embodied and enactive models such as *Subsumption* (Brooks, 1986) and *Predictive Processing* (Clark, 2013), and developmental frameworks inspired by infant cognition (Cangelosi and Schlesinger, 2015) like the *iCub Cognitive Architecture* (Vernon et al., 2007), the *Distributed Adaptive Control (DAC)* and *OpenCog* (Hart and Goertzel, 2008). While these architectures have provided valuable insights, many still rely on substantial pre-programmed structures, which can limit flexibility in open-ended, unsupervised developmental settings.

Furthermore, we consider two foundational conditions to be crucial for Artificial Cognition development: (i) experience as a continuous, situated, and agent-centered process; and (ii) the agent as an “altricial” entity, i.e., “designed” from the start to develop by relying not only on its own competences but rather on the interaction with other social, cognitive agents (Sloman and Chappell, 2005). In our view, experience is not a passive source of data, but it is the ongoing result of embodied interaction with the world, progressively internalized through memory, simulation, and prediction. Knowledge thus emerges from the perspective of a situated *“self,”* shaped by bodily constraints, environmental conditions, and opportunities for interaction (Incao et al., 2025). At the same time, cognition is not expected to emerge in isolation and only later become social, rather, it is scaffolded from the outset by an environment populated by intelligent others. Hence, we could draw inspiration from humans, where higher cognitive functions arise through prolonged embodied and social interaction rather than through pre-specified competence (Vygotsky, 1978).

In this manuscript, we investigate the minimal set of phylogenetic functions required to instantiate the autonomous, interactive acquisition and organization of knowledge. We conceive cognitive agents as dynamical systems of parallel loops evolving over time. Building on this view, we discuss the ontogenetic and epigenetic processes underlying the co-evolution of body structure and cognitive functions, and identify three key pillars: *embodiment*, *memory*, and *values*. Finally, we present our first implementation effort in this direction, the *iSEED (Interaction-oriented Situated Embodied Experience-Driven) cognitive architecture,* and discuss its future development and potential impact.

## 2 Cognition as a dynamical system of loops evolving through time

We argue that cognitive agents operate through the continuous interaction of multiple coupled dynamical loops (Figure 1). Behavior is not simply triggered by external requests or events (Quattrociocchi et al., 2025), but emerges from evolving exchanges among *internal states*, *embodied action in the physical world*, and *social interaction* (Kotseruba and Tsotsos, 2020; Laird et al., 1987), all supported by *memory*. In this view, cognition is not a single perception–action cycle, but *a set of interacting loops that progressively encode the relations between actions and their consequences across multiple time scales, combining causal structure with the statistical regularities of experience* (Anderson et al., 1997; Brooks, 1986). Fast loops capture immediate sensorimotor contingencies, whereas slower loops integrate experience, stabilize behavioral patterns, and support anticipation of delayed outcomes. Unlike dual-process theories such as the System 1/System 2 model (Kahneman, 2011), which assume a discrete number of distinct processing systems, we hypothesize that the foundational ability of cognitive agents is to generate, sustain, close, and make sense of multiple parallel loops. Rather than pairing a reactive- with a reasoning-oriented loop, we argue that through development, cognitive agents progressively expand, reuse and multiply these loops across broader bodily, physical, social, and temporal horizons.

**Figure 1:**
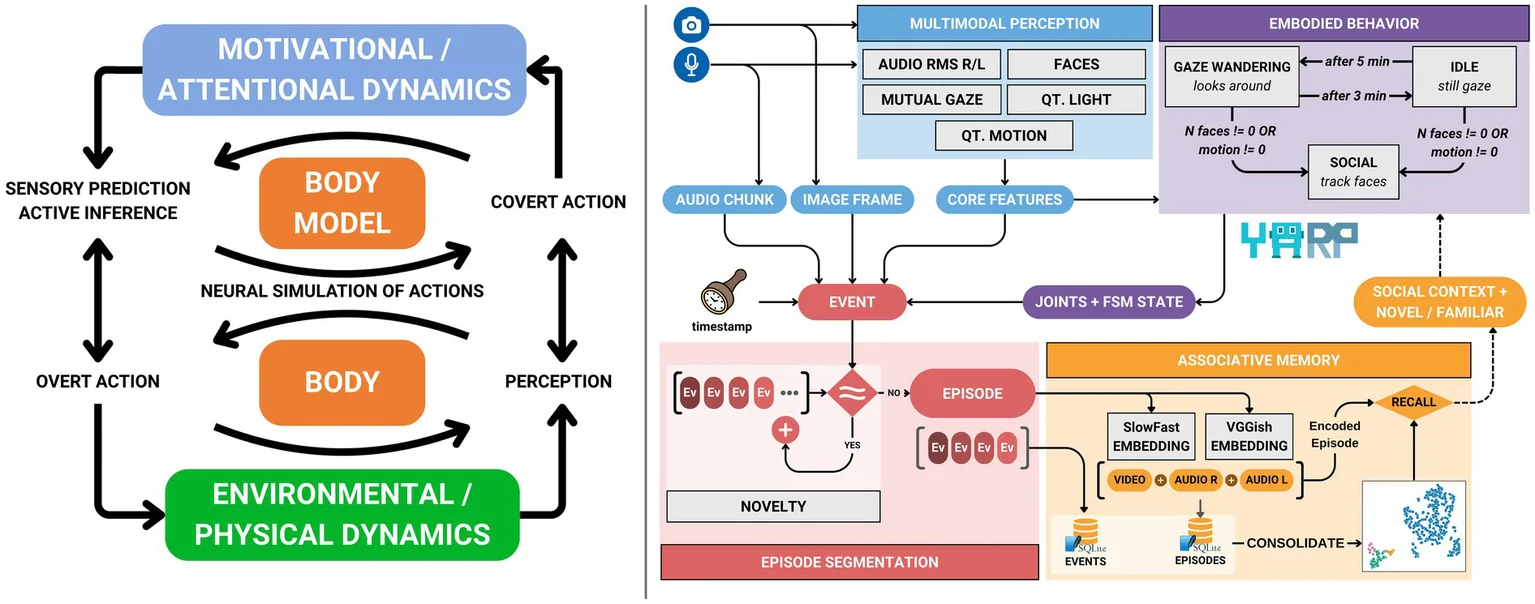
(left) cognition as a dynamical system with multiple parallel loops coupling the body, body model, internal and environmental dynamics. (right) iSEED cognitive architecture. See section 2.1 for more details on covert and overt actions.

From this perspective, the basic phylogenetic architecture should provide a mechanism for developing and closing the loops between the body and the environment (physical and social) in a prediction-exploration pattern (Sandini et al., 2024; Nagai, 2019). The same underlying hardware should support both action and internal simulation, enabling the agent to act, predict, and anticipate others using shared embodied mechanisms (Rizzolatti and Craighero, 2004). Through ontogenesis, this basic organization progressively expands, together with the agent’s sensorimotor and cognitive skills, allowing loops to span longer timescales and support the increasingly complex adaptive behavior of human adults. Since these loops are initially grounded in the body and in situated interaction (Filippetti, 2021), they are inherently tied to time and space. It is precisely their developmental expansion, together with memory and internal simulation, that enables the agent to remain anchored in the “here and now” while also becoming retrospective and predictive, through memory and anticipation.

For most AI and LLM-based systems, *time* is considered a background variable, with knowledge encoded as static parameters that carry little information about when it was acquired, under which conditions, or how it changed through interaction (Firoozi et al., 2025; Qiu et al., n.d.). In contrast, we view time as the epistemic roadmap through which knowledge and experience emerge (Incao et al., 2025; Smith and Thelen, 2003; Kärtner and Köster, 2024). Within this temporal framework, *embodiment, memory,* and *values* constitute three core pillars that enable the emergence and progressive stabilization of the cognitive loops. The *body* grounds cognition in embodied interaction; *memory* integrates experience across time into a coherent, evolving, and personalized structure; and *values* guide and give meaning to action by defining what is relevant, desirable, and worth pursuing.

### 2.1 Body and body model

Cognition does not emerge independently of the body, but through continuous coupling between bodily processes and interaction with the world (Sandini et al., 2025; Morasso, 2026; Barsalou, n.d.). Recent advances in Physical AI, Vision-Language-Action models, and world models increasingly strengthen sensorimotor grounding, cross-platform adaptation, and prediction of action consequences (Ma et al., 2026; Ding et al., 2025; Gaba et al., 2026). Nevertheless, much of their knowledge remains encoded in transferable parameters, designed to be reused across embodiments (Firoozi et al., 2025; Xu et al., 2025; Dove, n.d.). Although this enables scalability, it partially separates acquired knowledge from the bodily and environmental conditions under which it was formed (Barsalou, n.d.); potentially limiting its grounding in physical and social interaction (Firoozi et al., 2025; Dove, n.d.; Nathan, 2023). This differs from biological agents, whose knowledge develops through, and remains inseparable from, their particular embodiment and interaction history. Biological cognition therefore emerges from the co-development of body and mind as a coupled system. A minimal, phylogenetically constrained body develop alongside cognition through continuous interaction, producing knowledge that is inherently embodied, situated, and personal (Vernon, 2014). This coupling supports internal models of the body, environment, and other agents (Wolpert et al., 1998), enabling internal simulation and the anticipation of actions and events’ consequences (Rieger et al., 2024; Vicente et al., 2016). Behavior thus arises from the interplay between overt actions, executed in the world, and covert actions, internally simulated to evaluate possible outcomes, with first-hand experience continuously refining the underlying models. Cognition, in this sense, is not simply computed but enacted and grounded in the continuous coupling between body and world (Sandini et al., 2025; Buhrmann et al., 2013).

### 2.2 Memory

Memory gives cognition its temporal depth, enabling the agent to connect past experience with present behavior and future anticipation (Qiu et al., n.d.; Smith and Thelen, 2003). Without memory, cognition would remain limited to immediate perception–action contingencies, unable to close loops unfolding over longer temporal or conceptual scales (Kärtner and Köster, 2024; Morasso, 2026). Recent memory-augmented agents can retrieve previous interactions to guide current behavior and dynamically reorganize stored knowledge (Xu et al., 2025; Lee et al., 2017), while continual-learning methods increasingly allow robots to preserve and recombine capabilities across successive tasks (Meng et al., 2025). Nevertheless, these mechanisms are generally designed to improve contextual reasoning, task performance, or knowledge retention. In biological cognitive agents, memory additionally operates as an active and evolving developmental process, tightly coupled with ongoing interaction, internal state, and changing goals (Kärtner and Köster, 2024; Barsalou, n.d.). Experiences are therefore not retained as immutable records, but progressively encoded, reorganized, and reshaped over time (Barsalou, n.d.; Gaba et al., 2026). Since biological systems operate under finite capacity, they depend on consolidation, compression, reinforcement and decay, to transform short-term traces into long-term structures (Xu et al., 2025; Dove, n.d.). These representations are continuously updated by new experiences, goals and internal states, making long-term memory inherently reconstructive and plastic (Barsalou, n.d.; Nathan, 2023). Retrieval is equally active and constructive: past episodes are associated and reinterpreted in light of the current context and subsequent learning, allowing the same experience to acquire new meaning over time (Wolpert et al., 1998). In this way, memory enables the agent to establish causal links across distant events, interpret delayed outcomes (Morasso, 2026; Rieger et al., 2024), and support internal simulation where past experience is recombined to anticipate possible futures (Smith and Thelen, 2003; Vicente et al., 2016). Therefore, memory is not merely a resource for retaining and retrieving information, but a developmental process that gives cognition continuity, anticipation and meaning.

### 2.3 Values, motivation, goals and personality

Time, embodiment and memory allow cognition to develop and organize experience, but they do not by themselves determine how the agent behaves. This requires an additional layer of *values, motivation, goals and individual preferences*, defining what is relevant, desirable, and worth pursuing in the contingent situation (Buhrmann et al., 2013; Schacter et al., 2007). Recent agentic AI architectures already combine explicit goals, contextual memory, planning, reflection, and feedback-driven execution, while active-inference systems integrate prediction, action selection, and adaptive control (Bandi et al., 2025; Szadkowski and Faigl, 2025). Nevertheless, these capabilities are generally organized around externally specified objectives and evaluation criteria. Artificial constraints may reproduce value-consistent behavior, but they do not necessarily become endogenous values causally grounded in the agent’s own experience (Wu and Shu, n.d.; Schacter et al., 2012). Such systems can therefore evaluate the consequences of their actions and prioritize behavior only with respect to externally-defined objectives, without possessing an internal, developmentally-formed perspective from which outcomes acquire personal significance (Sandini et al., 2024; Morasso, 2026; Vatican, n.d.). Biological agents, by contrast, are guided by internal drives that evolve over development (Liu et al., 2022; Lee et al., 2017). Early behavior is shaped by regulatory needs and primitive value structures linked to survival and preservation, which already bias attention and interaction—e.g., toward social stimuli (Sridhar et al., 2023). Over time, these are complemented by intrinsic motivations such as curiosity and exploration, which drive engagement even in the absence of immediate reward – making the very act of exploring rewarding (Nagy et al., 2025; Ryan and Frankland, 2022). Repeated interaction with caregivers and peers, then shapes increasingly structured goals, norms, and beliefs, eventually giving rise to relatively stable, but revisable, preferences and dispositions, namely personality (Zhu et al., n.d.). Crucially, values and goals are tightly coupled with memory and internal simulation in a self-reinforcing loop (Buhrmann et al., 2013; Schacter et al., 2007). They influence which experiences are attended, reinforced, and remembered, while memory continually reshapes them through the reinterpretation of past events (Kärtner and Köster, 2024). Likewise, internal simulation depends on this evaluative layer: without values, predicted outcomes cannot be assessed or prioritized. Values thus allow the agent not only to predict what may happen, but to decide what should be pursued. In this sense, cognition is not only a matter of knowing, but of caring—i.e., assigning significance to outcomes. For robotics, developmentally grounded intrinsic drives may support the transition from externally directed behavior toward more autonomous, adaptive, and socially meaningful agents (Lee et al., 2017).

## 3 The iSEED cognitive architecture

Building on this perspective, we moved from theory to implementation. Within the *iCog Initiative*^1^—a series of multidisciplinary international workshops aimed at collaboratively design a cognitive architecture for the humanoid robot iCub—we developed iSEED (Interaction-oriented Situated Embodied Experience-Driven), an Always-On cognitive architecture intended as an experimental framework for investigating the minimal conditions under which personal, situated knowledge can emerge over time (Varela et al., 1991). Guided by Vernon’s definition of cognition as “*the process by which an autonomous system perceives, learns from experience, anticipates outcomes, acts toward goals, and adapts to changing circumstances*” (Vernon, 2014); we designed iSEED as an embodied agent that (i) continuously perceives its surroundings through multimodal sensing and (ii) incrementally organizes its own experience into emerging regularities (Pasquali et al., 2025a).

We embodied iSEED in the ICubHead—a 6-DoF actuated iCub robot head (Metta et al., n.d.), mounted on a stationary upper body and equipped with RGB cameras and an external stereo microphone—so the robot could remain continuously active, while interacting with the environment and progressively building its own ontogenetic knowledge. The system has no explicit task; instead, its behavior is driven by a curiosity-inspired proto-motivation, i.e., being attentive toward environmental deviations that disrupt perceptual consistency and may reveal socially meaningful patterns (Gottlieb et al., 2013). This design directly reflects the framework presented above: it supports continuous activity and developmental unfolding, it is grounded in an embodied perceptual loop, it incrementally encodes and consolidates personal experience, and it implements a minimal proto-motivational structure based on social saliency, novelty detection, and consistency maintenance.

In this sense, iSEED should not be seen as a complete cognitive architecture, but as a minimal testbed for studying the early emergence of Artificial Cognition. The architecture is currently organized into four modules.

The **Multimodal Perception** module processes real-time RGB video and audio, extracting five core features to which humans are sensitive since early development: number of faces (Farroni et al., 2002), *mutual gaze* (Reddy, 2003; Kaplan and Hafner, 2006), *motion* (Simion et al., 2008; Alegria and Noirot, 1978), *illumination, and stereo audio energy* (Alegria and Noirot, 1978). These features were selected as minimal anchors for social and environmental engagement. Perception is intentionally lightweight: the system does not yet distinguish speech from other sounds or assign semantic meaning to visual or auditory input. Instead, it tracks a compact set of developmentally relevant cues sufficient to scaffold interaction and regularity detection.

The **Embodied Behavior** module controls the ICubHead through alternating phases of stillness and gaze exploration, allowing the robot to seek and track human faces. In this minimal form of active perception, the body is not merely a passive sensor carrier but a mechanism for structuring experience. By orienting toward socially salient stimuli (Hofsten, 2009), the agent begins to couple internal activity with external regularities, laying the basis for the later emergence of richer body and social models.

The **Episode Segmentation** module links perception, embodiment, and temporal organization. First, it synchronizes sensory data and joint states into timestamped Event objects. Then, it groups events into Episodes, defined as temporally contiguous sequences sharing a stable context. Segmentation is driven by a *curiosity-inspired drive*: a new episode is created whenever one or more perceptual features deviate significantly from the statistics of the current episode (Gottlieb et al., 2013; Zacks and Swallow, 2007)—i.e., at least 2 times from the mean of each modality. Importantly, features are weighed differently, with changes in the number of faces carrying more saliency than motion or audio, reflecting a minimal proto-value system (Baldassarre et al., 2014). Segmentation therefore acts as an initial form of value-guided experience structuring.

Lastly, the **Associative Memory** provides four functions: episode encoding, consolidation, recognition, and temporal expectation. Each episode is encoded into a multimodal 528-long embedding combining the output of the SlowFast (Feichtenhofer et al., 2019) and VGGish (Hershey et al., 2017) pretrained models, together with summary statistics. During daily consolidations, the system reorganizes episodes into associative clusters through unsupervised dimensionality reduction (Modirshanechi et al., 2023) and density-based clustering (Malzer and Baum, 2020)^2^. These clusters act as emergent social contexts, enabling the agent to distinguish familiar from unfamiliar situations. Newly experienced episodes can then be compared to previously learned contexts, providing the agent with a minimal form of recognition, i.e., whether a situation is novel or resembles a known pattern. By aggregating these contexts over time windows, the architecture builds a primitive temporal expectation, estimating which type of context is likely to occur at a given time and identifying deviations from that expectation.

## 4 Experimental milestones

Before closing the action loop, allowing the agent to behave driven by experience and personal values, we evaluated whether (i) the iSEED architecture could learn relevant associations from unsupervised experience, and (ii) if these associations remain sufficiently stable over time to support recognition and expectation. We deployed the ICubHead, equipped with iSEED, in two socially rich environments: our open office at the Italian Institute of Technology (449 episode over 24 h across 4 days) and the demo session of an international conference (2,304 episodes over 15 h across 3 days). In both settings, the robot continuously observed human activity while orienting its gaze toward salient social stimuli. See (Pasquali et al., 2025a) for more details about the evaluation protocol and reproducibility details.

From the first days of observation, the architecture autonomously organized its experience into two distinct clusters (Pasquali et al., 2025a). Inspection of their content and statistical grounding showed that the system had learned to distinguish between lively episodes, characterized by higher levels of faces, mutual gaze, motion, and audio, and calm episodes, marked by little or no human activity (Shin and DuBrow, 2021). The result suggests that socially meaningful regularities can be extracted directly from first-person multimodal experience. Moreover, the associative structure remained coherent even when experiences from both environments were merged, indicating that the learned representations were both environment-sensitive and incrementally compositional (Pasquali et al., 2025a) (Table 1).

**Table 1:** Active hours and computed metrics from the IIT office and Conference validations.

	Office				Conference		
Metric	Day 0	Day 1	Day 2	Day 3	Day 0	Day 1	Day 2
Active hours	10 a.m.–4 p.m.				10–11 a.m., 2–5 p.m.	2–6 p.m.	9 a.m.–2 p.m.
Collected episodes	19	217	130	83	476	817	1,011
Eps. recognition accuracy	–	0.23	0.87	0.93	–	0.83	0.73
Temporal stability	0.61	0.76	0.79	–	0.79	0.77	–
Expectation consistency	–	0.24	0.95	0.96	–	0.26	0.08

Then, we assessed whether these emerging associations were stable enough over time to support prediction and expectation. However, we intentionally avoided imposing externally supervised labels; hence to validate the episode’s recognition and expectation, we aligned with the agent’s goal of preserving internal consistency. We evaluated (i) if episodes received the same context label in real-time and after daily consolidation (*recognition accuracy*); (ii) if the label remained consistent across consecutive days (*temporal stability*); and (iii) if the real-time context matched the one most likely to occur at that time based on prior experience (*expectation consistency*). In the office performance improved steadily with experience, suggesting that, in a moderately regular environment, iSEED can progressively form a stable associative structure that supports both recognition and temporal expectation. The conference showed a complementary pattern: the accuracy and stability remained reasonably good, while the expectation consistency was lower, likely due to the higher variability of the environment and the different recording schedules across days. These results highlight how the architecture’s performance depends on the regularity and predictability of the experienced environment, as expected for a memory grounded in first-person developmental experience rather than in pre-labeled abstractions.

These milestones provide initial evidence that iSEED can segment and encode multimodal experience, consolidate them into meaningful associative structures, and use those structures to recognize familiar contexts and infer simple temporal expectations. While this does not yet constitute full Artificial Cognition, it supports our core hypothesis: a minimal architecture grounded in continuous interaction, embodiment, and memory, and guided by simple proto-motivational mechanisms, can begin to organize personal experience into stable internal regularities.

## 5 Discussion and conclusion

In this paper, we outlined a roadmap to enable social robots to learn and adapt through development, acquired personal experience and associative memory, rather than relying solely on pre-specified competence. As a first implementation of this perspective, we introduced iSEED, showing that even a minimal cognitive architecture can instantiate several foundational ingredients of Artificial Cognition. ISEED conceptually builds around the three core pillars: *embodiment, memory*, and *values*. In particular, it supports continuous embodied perception through multimodal sensing and prosocial gaze behavior. Also, it autonomously segments ongoing interaction into meaningful episodes. Repeated first-person experiences give rise to associative regularities, which are progressively organized into coherent internal representations. Lastly, it exhibits early forms of context recognition and temporal expectation, allowing the agent to anticipate recurring social dynamics.

At the same time, iSEED remains a preliminary implementation of our broader perspective, a small step in a long journey toward our long-term research objective of building genuinely adaptive, socially meaningful, and personally situated embodied agents. Our first next step will be to move from the current proof of concept toward modules endowed with their own intrinsic temporal dynamics. The current implementation still involves a sequential processing of perception, segmentation, memorization and recognition meant to validate the basic organization of experience. However, a fuller realization of the proposed dynamical-loops framework requires these processes to unfold continuously and interact over multiple timescales. Perceptual dynamics should remain tightly coupled with the ongoing evolution of the external world, while memory and motivational dynamics may follow different temporal profiles, such as consolidation, reinforcement, decay, or gradual changes in saliency.

In parallel, we are enabling the iSEED to close the cognitive loops, i.e., to decide how to act given the perceived context, past experiences and the agent values. To this end, several components should be extended. Context recognition must become continuous and online, rather than only available after episode completion, enabling the agent to maintain an updated representation while interaction is taking place. Contextual labels, along with the agent’s experience and internal values, should drive the robot to behave consistently and proactively toward its own goals. To study these next steps, we are also exploring a complementary paradigm: “The Robotic Nursery”, a developmentally inspired environment, such as a floor playmat or a high-chair setup, in which an artificial agent can progressively acquire richer sensorimotor, social, and contextual experience, under controlled but naturalistic conditions.

### 5.1 Impact on robotics

More broadly, the novel perspective proposed in this manuscript challenges the current rush toward increasingly general-purpose robotic intelligence, in which a single pretrained model is expected to provide a universal solution across tasks, environments and users. While this vision is technologically compelling, it risks overlooking what many real-world robotic applications actually require: not a one-size-fits-all agent, but a personal, situated, embodied agent able to incrementally learn the specific regularities, needs and social dynamics of the context in which it operates. In homes, clinics, schools and workplaces, effective behavior strongly depends on the ability to adapt to specific people, routines, expectations and exceptions through continuous embodied interaction. A developmental architecture allows competence to emerge locally from first-hand experience rather than being solely fully specified in advance through massive pretraining. Such an approach could yield robots that are more robust to context variability, more sensitive to individual users, more teachable through everyday interaction, and less dependent on centralized data collection and repeated training. Certainly, growing all the robots from scratch is technically unfeasible. Hence, the long-term goal is not to replace pretraining altogether, but to see it as a sort of *“evolutionary background”* purposely bootstrapping always-on developmental mechanisms that allow robots to be genuinely adaptive, socially meaningful, and personally situated agents. From this perspective, the real challenge for robotics is not to build agents that know everything in advance, but agents that can progressively become what their specific social environment requires them to be.

## Data Availability

The raw data supporting the conclusions of this article will be deposited in a restricted-access Zenodo repository. Access to the data will be provided by the authors upon reasonable request, without undue reservation.

## Footnotes

1
https://icog.eu/

2 Hyperparameters for UMAP {METRIC: EUCLIDEAN, MIN_DIST: 0.0, N_COMPONENTS: 3, N_NEIGHBORS: 10} and HDBSCAN {MIN_CLUSTER_SIZE: 5, MIN_SAMPLES: 50}.

## References

[ref1] Agarwal S. “Thousands of People are selling their Identities to train AI – but at what cost?,” The Guardian. (2026). Available online at: https://www.theguardian.com/technology/2026/mar/21/ai-trainers-identity-cost

[ref2] Anaya B. Pérez-Edgar K. (2019). Personality development in the context of individual traits and parenting dynamics. New Ideas Psychol. 53, 37–46. doi: 10.1016/j.newideapsych.2018.03.002, 30872892 PMC6411068

[ref3] Anderson J. R. Matessa M. Lebiere C. (1997). ACT-R: a theory of higher level cognition and its relation to visual attention. Hum. Comput. Interact. 12, 439–462. doi: 10.1207/s15327051hci1204_5

[ref4] Balconi M. Angioletti L. Acconito C. (2023). Self-awareness of goals task (SAGT) and planning skills: the neuroscience of decision making. Brain Sci. 13:1163. doi: 10.3390/brainsci13081163, 37626519 PMC10452128

[ref5] Baldassarre G. Stafford T. Mirolli M. Redgrave P. Ryan R. M. Barto A. (2014). Intrinsic motivations and open-ended development in animals, humans, and robots: an overview. Front. Psychol. 5:985. doi: 10.3389/fpsyg.2014.00985, 25249998 PMC4158798

[ref6] Bandi A. Kongari B. Naguru R. Pasnoor S. Vilipala S. V. (2025). The rise of agentic AI: a review of definitions, frameworks, architectures, applications, evaluation metrics, and challenges. Future Internet 17:404. doi: 10.3390/fi17090404

[ref7] Barsalou L. W. Challenges and opportunities for grounding cognition. J. Cogn. 3:31. doi: 10.5334/joc.116, 33043241 PMC7528688

[ref8] Biderman N. Bakkour A. Shohamy D. (2020). What are memories for? The hippocampus bridges past experience with future decisions. Trends Cogn. Sci. 24, 542–556. doi: 10.1016/j.tics.2020.04.004, 32513572

[ref9] Brooks R. (1986). A robust layered control system for a mobile robot. IEEE J. Robot. Autom. 2, 14–23. doi: 10.1109/JRA.1986.1087032

[ref10] Buhrmann T. Di Paolo E. A. Barandiaran X. (2013). A dynamical systems account of sensorimotor contingencies. Front. Psychol. 4:285. doi: 10.3389/fpsyg.2013.00285, 23750143 PMC3664438

[ref11] Cangelosi A. Schlesinger M. (2015). Developmental Robotics: From Babies to Robots. The MIT Press.

[ref12] Clark A. (2013). Whatever next? Predictive brains, situated agents, and the future of cognitive science. Behav. Brain Sci. 36, 181–204. doi: 10.1017/S0140525X12000477 23663408

[ref13] Cowan E. T. Schapiro A. C. Dunsmoor J. E. Murty V. P. (2021). Memory consolidation as an adaptive process. Psychon. Bull. Rev. 28, 1796–1810. doi: 10.3758/s13423-021-01978-x, 34327677

[ref14] Criscuolo A. Schwartze M. Kotz S. A. (2022). Cognition through the lens of a body–brain dynamic system. Trends Neurosci. 45, 667–677. doi: 10.1016/j.tins.2022.06.004, 35810022

[ref15] Ding J. Zhang Y. Shang Y. Zong Z. Feng J. Yuan Y. . (2025). Understanding world or predicting future? A comprehensive survey of world models. ACM Comput. Surv. 58, 1–38. doi: 10.1145/3746449

[ref16] Dove G. Symbol ungrounding: what the successes (and failures) of large language models reveal about human cognition. Philos. Trans. R. Soc. B Biol. Sci. 379:20230149. doi: 10.1098/rstb.2023.0149 PMC11529626 39155725

[ref17] Farroni T. Csibra G. Simion F. Johnson M. H. (2002). Eye contact detection in humans from birth. Proc. Natl. Acad. Sci. USA 99, 9602–9605. doi: 10.1073/pnas.152159999, 12082186 PMC123187

[ref18] Feichtenhofer C. Fan H. Malik J. He K. (2019). “SlowFast Networks for Video Recognition,” in 2019 IEEE/CVF International Conference on Computer Vision (ICCV). Seoul, Korea (South): IEEE, 6201–6210 doi: 10.1109/ICCV.2019.00630.

[ref19] Filippetti M. L. (2021). Being in tune with your body: the emergence of interoceptive processing through caregiver–infant feeding interactions. Child Dev. Perspect. 15, 182–188. doi: 10.1111/cdep.12420

[ref20] Firoozi R. Tucker J. Tian S. Majumdar A. Sun J. Liu W. . (2025). Foundation models in robotics: applications, challenges, and the future. Int. J. Robot. Res. 44, 701–739. doi: 10.1177/02783649241281508

[ref21] Gaba S. Rana K. Sai S. Chamola V. Niyato D. (2026). A comprehensive review of physical artificial intelligence. doi: 10.36227/techrxiv.176739762.23746519/v1

[ref22] Gottlieb J. Oudeyer P.-Y. Lopes M. Baranes A. (2013). Information-seeking, curiosity, and attention: computational and neural mechanisms. Trends Cogn. Sci. 17, 585–593. doi: 10.1016/j.tics.2013.09.001, 24126129 PMC4193662

[ref23] Hart D. Goertzel B. (2008). “OpenCog: a software framework for integrative artificial general intelligence,” in Artificial General Intelligence 2008, (The Netherlands: OS Press / Sage Publishing Teleportboulevard 120 1043 EJ Amsterdam), 468–472. https://ebooks.iospress.nl/publication/4111

[ref24] Hershey S. . (2017). CNN architectures for large-scale audio classification. arXiv: arXiv:1609.09430. http://arxiv.org/abs/1609.09430

[ref25] Hofsten C. V. (2009). Action, the foundation for cognitive development. Scand. J. Psychol. 50, 617–623. doi: 10.1111/j.1467-9450.2009.00780.x, 19930261

[ref26] Ilyka D. Johnson M. H. Lloyd-Fox S. (2021). Infant social interactions and brain development: a systematic review. Neurosci. Biobehav. Rev. 130, 448–469. doi: 10.1016/j.neubiorev.2021.09.001, 34506843 PMC8522805

[ref27] Incao S. Sciutti A. Gallagher S. (2025). Robodiment, self and temporality: phenomenological insights for cognitive architectures in robotics. Minds Mach. 35:49. doi: 10.1007/s11023-025-09750-z

[ref28] Alegria J. Noirot E. (1978). Neonate orientation behaviour towards human voice. J.Alegria, E.Noirot. doi: 10.1177/016502547800100401 24923609

[ref29] Kadambi A. Aziz-Zadeh L. Damasio A. Iacoboni M. Narayanan S. (2026). Embodiment in multimodal large language models. Neuron 114, 1908–1921. doi: 10.1016/j.neuron.2026.03.004, 41928508

[ref30] Kahneman D. (2011). Thinking, Fast and Slow. Farrar, Straus and Giroux.

[ref31] Kaplan F. Hafner V. V. (2006). The challenges of joint attention. Interact. Stud. 7, 135–169. doi: 10.1075/is.7.2.04kap

[ref32] Kärtner J. Köster M. (2024). Early social-cognitive development as a dynamic developmental system—a lifeworld approach. Front. Psychol. 15:1399903. doi: 10.3389/fpsyg.2024.1399903, 38939231 PMC11210372

[ref33] Kharpal A. “Surging AI demand Could cause the World’s next chip Shortage, Research Says,” CNBC. Available online at: https://www.cnbc.com/2024/09/25/surging-ai-demand-could-cause-the-worlds-next-chip-shortage-report.html

[ref34] Kotseruba I. Tsotsos J. K. (2020). 40 years of cognitive architectures: core cognitive abilities and practical applications. Artif. Intell. Rev. 53, 17–94. doi: 10.1007/s10462-018-9646-y

[ref35] Laird J. E. Newell A. Rosenbloom P. S. (1987). SOAR: an architecture for general intelligence. Artif. Intell. 33, 1–64. doi: 10.1016/0004-3702(87)90050-6

[ref36] Lee J. L. C. Nader K. Schiller D. (2017). An update on memory reconsolidation updating. Trends Cogn. Sci. 21, 531–545. doi: 10.1016/j.tics.2017.04.006, 28495311 PMC5605913

[ref37] Leisman G. Roy R. Alfasi R. (2026). Perception as self-organizing interaction: embodied cognition, artificial intelligence, and autism. Front. Psychol. 17:1803234. doi: 10.3389/fpsyg.2026.1803234, 41982360 PMC13070933

[ref38] Liu W. Shi Y. Cousins J. N. Kohn N. Fernández G. (2022). Hippocampal-medial prefrontal Event segmentation and integration contribute to episodic memory formation. Cereb. Cortex 32, 949–969. doi: 10.1093/cercor/bhab258, 34398213

[ref39] Liu V. Yin Y. (2024). Green AI: exploring carbon footprints, mitigation strategies, and trade offs in large language model training. Discov. Artif. Intell. 4:49. doi: 10.1007/s44163-024-00149-w

[ref40] Ma Y. Song Z. Zhuang Y. Hao J. King I. (2026). A survey on vision--language--action models for embodied AI. IEEE Trans. Neural Netw. Learn. Syst. 37, 3031–3051. doi: 10.1109/TNNLS.2025.3650584, 42048203

[ref41] Malzer C. Baum M. (2020). “A hybrid approach to hierarchical density-based cluster selection,” in 2020 IEEE International Conference on Multisensor Fusion and Integration for Intelligent Systems(MFI), 223–228.

[ref42] McInnes L. Healy J. Melville J. (2020). UMAP: uniform manifold approximation and projection for dimension reduction. arXiv: arXiv:1802.03426. http://arxiv.org/abs/1802.03426

[ref43] Meng Y. Bing Z. Yao X. Chen K. Huang K. Gao Y. . (2025). Preserving and combining knowledge in robotic lifelong reinforcement learning. Nat. Mach. Intell. 7, 256–269. doi: 10.1038/s42256-025-00983-2

[ref44] Metta G. Sandini G. Vernon D. Natale L. Nori F. “The iCub Humanoid Robot,” in 8th Workshop on Performance Metrics for Intelli-gent Systems, 50.

[ref45] Modirshanechi A. Kondrakiewicz K. Gerstner W. Haesler S. (2023). Curiosity-driven exploration: foundations in neuroscience and computational modeling. Trends Neurosci. 46, 1054–1066. doi: 10.1016/j.tins.2023.10.002, 37925342

[ref46] Mon-Williams R. Li G. Long R. Du W. Lucas C. G. (2025). Embodied large language models enable robots to complete complex tasks in unpredictable environments. Nat. Mach. Intell. 7, 592–601. doi: 10.1038/s42256-025-01005-x, 40391151 PMC12088599

[ref47] Morasso P. (2026). Bio-inspired cognitive robotics vs. embodied AI for socially acceptable, civilized robots. Front. Robot. AI 13:1714310. doi: 10.3389/frobt.2026.1714310, 41607863 PMC12834747

[ref48] Nagai Y. (2019). Predictive learning: its key role in early cognitive development. Philos. Trans. R. Soc. B Biol. Sci. 374:20180030. doi: 10.1098/rstb.2018.0030, 30852990 PMC6452246

[ref49] Nagy D. G. Orbán G. Wu C. M. (2025). Adaptive compression as a unifying framework for episodic and semantic memory. Nat. Rev. Psychol. 4, 484–498. doi: 10.1038/s44159-025-00458-6

[ref50] Nathan M. J. (2023). Disembodied AI and the limits to machine understanding of students’ embodied interactions. Front. Artif. Intell. 6:1148227. doi: 10.3389/frai.2023.1148227, 36937707 PMC10020609

[ref51] NVIDIA, . (2025). GR00T N1: an open foundation model for generalist humanoid robots. arXiv: arXiv:2503.14734. doi: 10.48550/arXiv.2503.14734

[ref52] Park J. S. Gollapudi K. Ke J. Nau M. Pappas I. Leong Y. C. (2026). Emotional arousal enhances narrative memories through functional integration of large-scale brain networks. Nat. Hum. Behav. 10, 370–383. doi: 10.1038/s41562-025-02315-1, 41083729

[ref53] Pasquali D. . (2025a). “No Robot is an Island: An Always-On Cognitive Architecture for Social Context Awareness in Dynamic Environments,” in 2025 IEEE International Conference on Development and Learning (ICDL), 1–8.

[ref54] Pasquali D. (2025b). No robot is an island: an always-on cognitive architecture for social context awareness in dynamic environments. Zenodo. Available online at: https://zenodo.org/records/15551864

[ref55] Patel S. P. McCurdy M. P. Frankenstein A. N. Sklenar A. M. Urban Levy P. Szpunar K. K. . (2022). The reciprocal relationship between episodic memory and future thinking: how the outcome of predictions is subsequently remembered. Brain Behav. 12:e2603. doi: 10.1002/brb3.2603, 36000544 PMC9480898

[ref56] Poli F. O’Reilly J. X. Mars R. B. Hunnius S. (2024). Curiosity and the dynamics of optimal exploration. Trends Cogn. Sci. 28, 441–453. doi: 10.1016/j.tics.2024.02.001, 38413257

[ref57] Qiu Y. Zhao Z. Ziser Y. Korhonen A. Ponti E. M. Cohen S. B. (n.d.). Are Large Language Models Temporally Grounded?.

[ref58] Quattrociocchi W. Capraro V. Perc M. (2025). Epistemological fault lines between human and artificial intelligence. arXiv: arXiv:2512.19466. doi: 10.48550/arXiv.2512.19466

[ref59] Raptis E. K. Kapoutsis A. C. C. Kosmatopoulos E. B. (2025). Agentic LLM-based robotic systems for real-world applications: a review on their agenticness and ethics. Front. Robot. AI 12. doi: 10.3389/frobt.2025.1605405, 40904652 PMC12402697

[ref60] Ray P. P. (2025). Physical AI: bridging the sim-to-real divide toward embodied, ethical, and autonomous intelligence. Mach. Learn. Comput. Sci. Eng. 2:1. doi: 10.1007/s44379-025-00050-y

[ref61] Rayyes R. (2023). Intrinsic motivation learning for real robot applications. Front. Robot. AI 10:1102438. doi: 10.3389/frobt.2023.1102438, 36845331 PMC9950409

[ref62] Reddy V. (2003). On being the object of attention: implications for self–other consciousness. Trends Cogn. Sci. 7, 397–402. doi: 10.1016/S1364-6613(03)00191-8, 12963470

[ref63] Rieger M. Boe S. G. Ingram T. G. J. Bart V. K. E. Dahm S. F. (2024). A theoretical perspective on action consequences in action imagery: internal prediction as an essential mechanism to detect errors. Psychol. Res. 88, 1849–1858. doi: 10.1007/s00426-023-01812-0, 36961546 PMC7616356

[ref64] Rizzolatti G. Craighero L. (2004). The mirror-neuron system. Annu. Rev. Neurosci. 27, 169–192. doi: 10.1146/annurev.neuro.27.070203.144230, 15217330

[ref65] Ryan T. J. Frankland P. W. (2022). Forgetting as a form of adaptive engram cell plasticity. Nat. Rev. Neurosci. 23, 173–186. doi: 10.1038/s41583-021-00548-3, 35027710

[ref66] Sandini G. Sciutti A. Morasso P. (2024). Artificial cognition vs. artificial intelligence for next-generation autonomous robotic agents. Front. Comput. Neurosci. 18:1349408. doi: 10.3389/fncom.2024.1349408, 38585280 PMC10995397

[ref67] Sandini G. Sciutti A. Morasso P. (2025). Mutual human-robot understanding for a robot-enhanced society: the crucial development of shared embodied cognition. Front. Artif. Intell. 8:1608014. doi: 10.3389/frai.2025.1608014, 40678782 PMC12269773

[ref68] Schacter D. L. Addis D. R. Buckner R. L. (2007). Remembering the past to imagine the future: the prospective brain. Nat. Rev. Neurosci. 8, 657–661. doi: 10.1038/nrn2213, 17700624

[ref69] Schacter D. L. Addis D. R. Hassabis D. Martin V. C. Spreng R. N. Szpunar K. K. (2012). The future of memory: remembering, imagining, and the brain. Neuron 76, 677–694. doi: 10.1016/j.neuron.2012.11.001, 23177955 PMC3815616

[ref70] Shin Y. S. DuBrow S. (2021). Structuring memory through inference-based event segmentation. Top. Cogn. Sci. 13, 106–127. doi: 10.1111/tops.12505, 32459391

[ref71] Simion F. Regolin L. Bulf H. (2008). A predisposition for biological motion in the newborn baby. Proc. Natl. Acad. Sci. 105, 809–813. doi: 10.1073/pnas.0707021105, 18174333 PMC2206618

[ref72] Simon E. “‘A different set of rules’: thermal drone Footage Shows Musk’s AI power plant Flouting clean air Regulations,” The Guardian, (2026). Available online at: https://www.theguardian.com/environment/2026/feb/13/elon-musk-xai-datacenters-air-pollution-mississippi

[ref73] Sloman A. Chappell J. (2005). The Altricial-Precocial Spectrum for robots: 19th international joint conference on artificial intelligence (Ijcai-05). Int. Jt. Conf. Artif. Intell 19, 1187–1192.

[ref74] Smith L. B. Thelen E. (2003). Development as a dynamic system. Trends Cogn. Sci. 7, 343–348. doi: 10.1016/S1364-6613(03)00156-6, 12907229

[ref75] Sridhar S. Khamaj A. Asthana M. K. (2023). Cognitive neuroscience perspective on memory: overview and summary. Front. Hum. Neurosci. 17:1217093. doi: 10.3389/fnhum.2023.1217093, 37565054 PMC10410470

[ref76] Szadkowski R. Faigl J. (2025). Lifelong active inference of gait control. IEEE Trans. Neural Netw. Learn. Syst. 36, 19133–19144. doi: 10.1109/TNNLS.2025.3579814, 40569806

[ref77] Varela F. J. Thompson E. Rosch E. (1991). “The embodied mind: cognitive science and human experience,” in The Embodied Mind: Cognitive Science and Human Experience, (Cambridge, MA, US: The MIT Press).

[ref78] Vatican (n.d.). Encyclical Letter of His Holiness Leo XIV Magnifica Humanitas. Available online at: http://www.vatican.va/content/leo-xiv/en/encyclicals/documents/20260515-magnifica-humanitas.html

[ref79] Vernon D. (2014). Artificial Cognitive Systems: A Primer. Cambridge, Massachusetts: The MIT Press.

[ref80] Vernon D. Metta G. Sandini G. (2007). “The iCub cognitive architecture: Interactive development in a humanoid robot,” in 2007 IEEE 6th International Conference on Development and Learning, 122–127.

[ref81] Vicente P. Jamone L. Bernardino A. (2016). Online Body Schema Adaptation Based on Internal Mental Simulation and Multisensory Feedback. Front. Robot. 3:7. doi: 10.3389/frobt.2016.00007

[ref82] Vygotsky L. S. (1978). Mind in Society: Development of Higher Psychological Processes. Cambridge, Massachusetts: Harvard University Press.

[ref83] Wolpert D. M. Miall R. C. Kawato M. (1998). Internal models in the cerebellum. Trends Cogn. Sci. 2, 338–347. doi: 10.1016/S1364-6613(98)01221-2, 21227230

[ref84] Wu P. Escontrela A. Hafner D. Abbeel P. Goldberg K. (2023). “DayDreamer: World Models for Physical Robot Learning,” in Proceedings of The 6th Conference on Robot Learning, (Auckland, New Zealand: PMLR), 2226–2240.

[ref85] Wu S. Shu K., “Memory in LLM-based Multi-agent Systems: Mechanisms, Challenges, and Collective Intelligence”.

[ref86] X, Email, and LinkedIn. “Why Hundreds of People in L.A. Are Strapping Cameras on Their Bodies to Do Chores,” Los Angeles Times. (2026). Available online at: https://www.latimes.com/business/story/2026-03-12/why-people-in-la-are-strapping-cameras-on-their-bodies-to-do-chores

[ref87] Xu W. Liang Z. Mei K. Gao H. Tan J. Zhang Y. (2025). A-Mem: Agentic Memory for LLM Agents Advances in Neural Information Processing Systems. Curran Associates, Inc., 17577–17604.

[ref88] Xu Q. Peng Y. Nastase S. A. Chodorow M. Wu M. Li P. (2025). Large language models without grounding recover non-sensorimotor but not sensorimotor features of human concepts. Nat. Hum. Behav. 9, 1871–1886. doi: 10.1038/s41562-025-02203-8, 40468013 PMC12454144

[ref89] Zacks J. M. Swallow K. M. (2007). Event segmentation. Curr. Dir. Psychol. Sci. 16, 80–84. doi: 10.1111/j.1467-8721.2007.00480.x, 22468032 PMC3314399

[ref90] Zhu Y. Zeng Y. Ren J. Zhang L. Chen C. Fernandez G. . Emotional learning retroactively promotes memory integration through rapid neural reactivation and reorganization. eLife 11:e60190. doi: 10.7554/eLife.60190, 36476501 PMC9815824

